# The search, coagulation, and clipping (SCC) method prevents delayed bleeding after gastric endoscopic submucosal dissection

**DOI:** 10.1007/s10120-018-0878-y

**Published:** 2018-09-28

**Authors:** Motoi Azumi, Manabu Takeuchi, Youhei Koseki, Masaru Kumagai, Yoko Kobayashi, Masafumi Takatsuna, Aiko Yoshioka, Seiichi Yoshikawa, Tsutomu Miura, Shuji Terai

**Affiliations:** 1 Division of Gastroenterology and Hepatology, Nagaoka Red Cross Hospital, 2-297-1, Chiaki, Nagaoka, Niigata 940-2085 Japan; 2 Division of Gastroenterology and Hepatology, Graduate School of Medical and Dental Sciences, Niigata University, Niigata, Japan

**Keywords:** Gastric endoscopic submucosal dissection, Delayed bleeding, Clip

## Abstract

**Background:**

Delayed bleeding is an important complication after gastric endoscopic submucosal dissection (ESD). The search, coagulation, and clipping (SCC) method can be used to prevent delayed bleeding after ESD. However, its safety and efficacy are unclear. We compared the SCC method with post-ESD coagulation (PEC) to clarify the safety and efficacy of the SCC method for preventing delayed bleeding after gastric ESD.

**Methods:**

This retrospective study included 438 patients (478 lesions) who underwent gastric ESD. Multivariate logistic regression analysis was performed to identify the significant independent factors associated with delayed bleeding and we performed propensity-score matching (PSM) to reduce the effect of procedure-selection bias of SCC method.

**Results:**

Of the 438 patients, 216 underwent PEC and 222 underwent SCC. Delayed bleeding was significantly less common in the SCC than in the PEC (2.6% vs. 7.2%; *P* = 0.013). Among patients treated with antithrombotic therapy, the delayed bleeding rate was lower in the SCC group than in the PEC group; however, the difference was not significant (*P* = 0.15). The SCC method was found to be a significant independent factor for the prevention of delayed bleeding. PSM was performed in 156 patients in the PEC group and SCC group. There was a significant difference in the incidence of bleeding in the PEC and SCC groups (*P* = 0.013). No patient had perforation/bleeding associated with the SCC method.

**Conclusions:**

Our findings suggest that the SCC method is a simple, safe, and effective approach for preventing delayed bleeding after gastric ESD.

## Introduction

Gastric endoscopic submucosal dissection (ESD) is widely used as a minimally invasive treatment option with a high cure rate for early gastric cancer that is limited to the intramucosal layer of the stomach. It is a first-line treatment approach for cancer involving very few lymph node metastases, particularly intramucosal carcinoma [1, 2]. However, delayed bleeding is one of the main postoperative complications. This complication can sometimes cause shock that might require transfusion. Thus, efforts should be made for its prevention, and a safe, simple, and cost-effective prevention method should be developed. Post-ESD coagulation (PEC) involving prophylactic cauterization of visible blood vessels at the ulcer floor is widely performed to prevent delayed bleeding after endoscopic therapy. The bleeding rate with this approach has been reported to be 3–5.5% [3–9]. Mukai et al. mapped pre-coagulated blood vessels and blood vessels with arterial bleeding during ESD and reported on the clipping of blood vessels mapped after PEC (coagulation plus artery-selective clipping; 2C method). They mentioned that the bleeding rate was low, with bleeding in only 1 of 80 cases [10]. The search, coagulation, and clipping (SCC) method, which involves observing the ulcer floor, identifying blood vessels, and cauterizing and clipping respective blood vessels, appears to be a good approach to prevent bleeding after ESD. However, the safety and efficacy of the SCC method are unclear. The present study aimed to clarify the safety and efficacy of the SCC method for preventing delayed bleeding after gastric ESD.

## Patients and methods

### Patients and data collection

This retrospective study included 438 patients (478 lesions) who underwent gastric ESD at Nagaoka Red Cross Hospital between January 1, 2014 and December 31, 2017. We reviewed the medical records and collected the following information: age at the time of ESD, sex, status of treatment with antithrombotic agents such as warfarin and antiplatelet drugs, gross endoscopic findings of the lesion, main seat of the lesion, time of ESD implementation, prophylactic treatment for delayed bleeding (PEC or SCC), number of clips, ESD complications, presence of delayed bleeding, resected specimen length, tumor length, histological tumor type, invasion depth, horizontal margin, vertical margin, presence of ulcer complications, presence of lymph node invasion, presence of venous invasion, procedures at the second look, and presence or absence of heparin placement. In patients who underwent ESD for multiple lesions, the lesion with the largest resection diameter was used for analysis. The medical costs were calculated using the amounts mentioned in the actual certificates of medical remuneration (medical bills), which were determined according to the Japanese Diagnosis Procedure Combination (DPC). All calculations were based on the currency conversion rate of 1 USD = 110.7 JPY as on May 24, 2018. This study was approved by the ethics committee of Nagaoka Red Cross hospital (No. 497).

### ESD procedure and management after ESD

ESD was performed using an IT Knife2 (KD-611L; Olympus, Tokyo, Japan) or a Hook Knife (KD-1L-1; Olympus). GIF-H260Z (Olympus) was used for assessing the lesion range and preoperative marking. For ESD, we used GIF-260J (Olympus) and a high-frequency generator (VIO300D; ERBE, Tubingen, Germany). A Coagrasper (FD-411QR; Olympus) was used for blood vessel cauterization during ESD or after ESD. After ESD, a proton-pump inhibitor (omeprazole 20 mg) was intravenously injected twice a day before resumption of oral food intake. A second-look endoscopic examination was performed on day 2, and on identifying bleeding or exposed blood vessels, hemostasis was achieved by cauterizing the blood vessels with a Coagrasper (FD-411QR; Olympus) or by clipping with hemostatic clips (HX-610-135; Olympus). If no issues were noted, oral food intake was resumed on day 2 or 3. Thereafter, oral esomeprazole (20 mg/day) or vonoprazan (20 mg/day) was administered for a minimum of 8 weeks [11, 12].

### SCC method

**Fig. 1:**
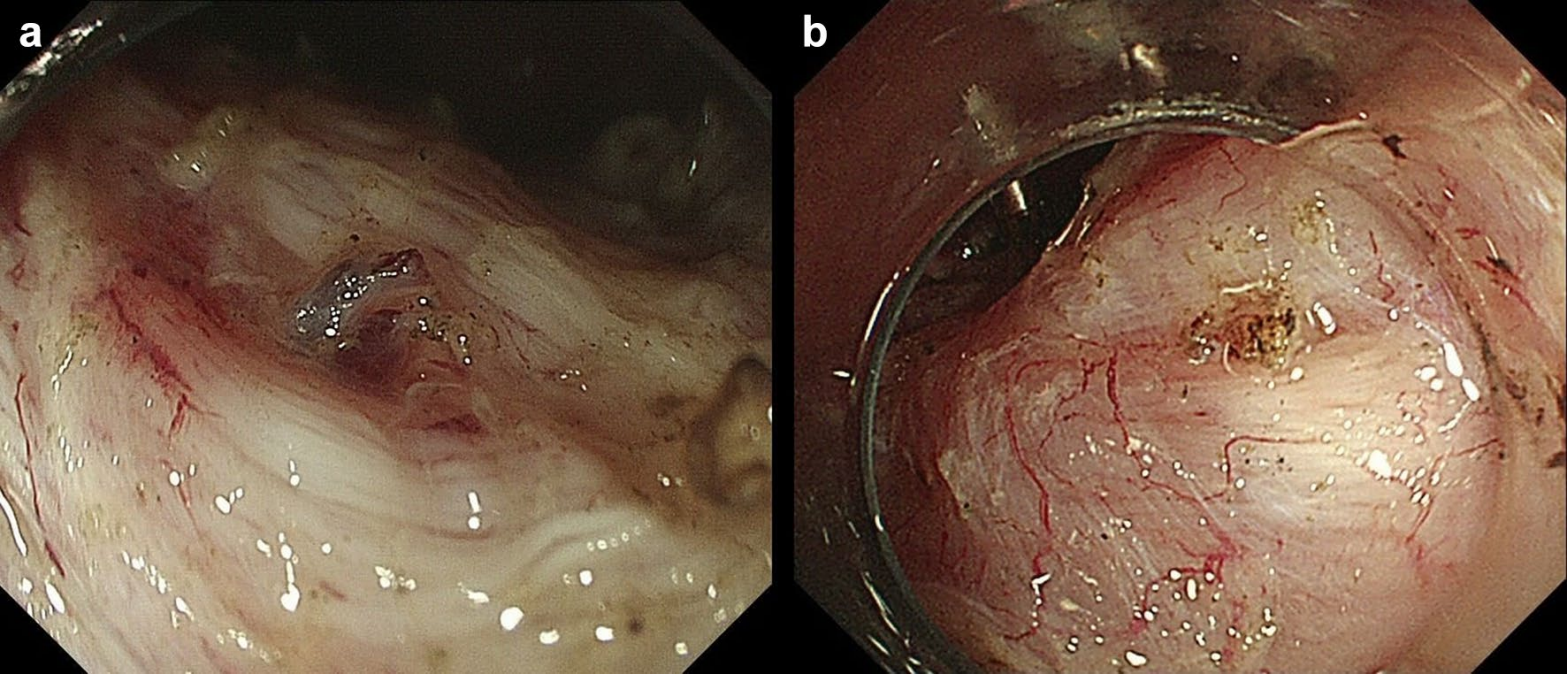
**a** Blood vessel pre-coagulated during ESD is observed. It was thought to be a penetrating branch passing through the muscle layer. Thus, a clip was placed. **b** Blood vessel could not be identified during ESD but could be identified on observation using GIF-H260Z (Olympus, Tokyo, Japan). The blood vessel is small, but a pulse was detected. Thus, it was diagnosed as an artery, and a clip was placed. *ESD* endoscopic submucosal dissection

**Fig. 2:**
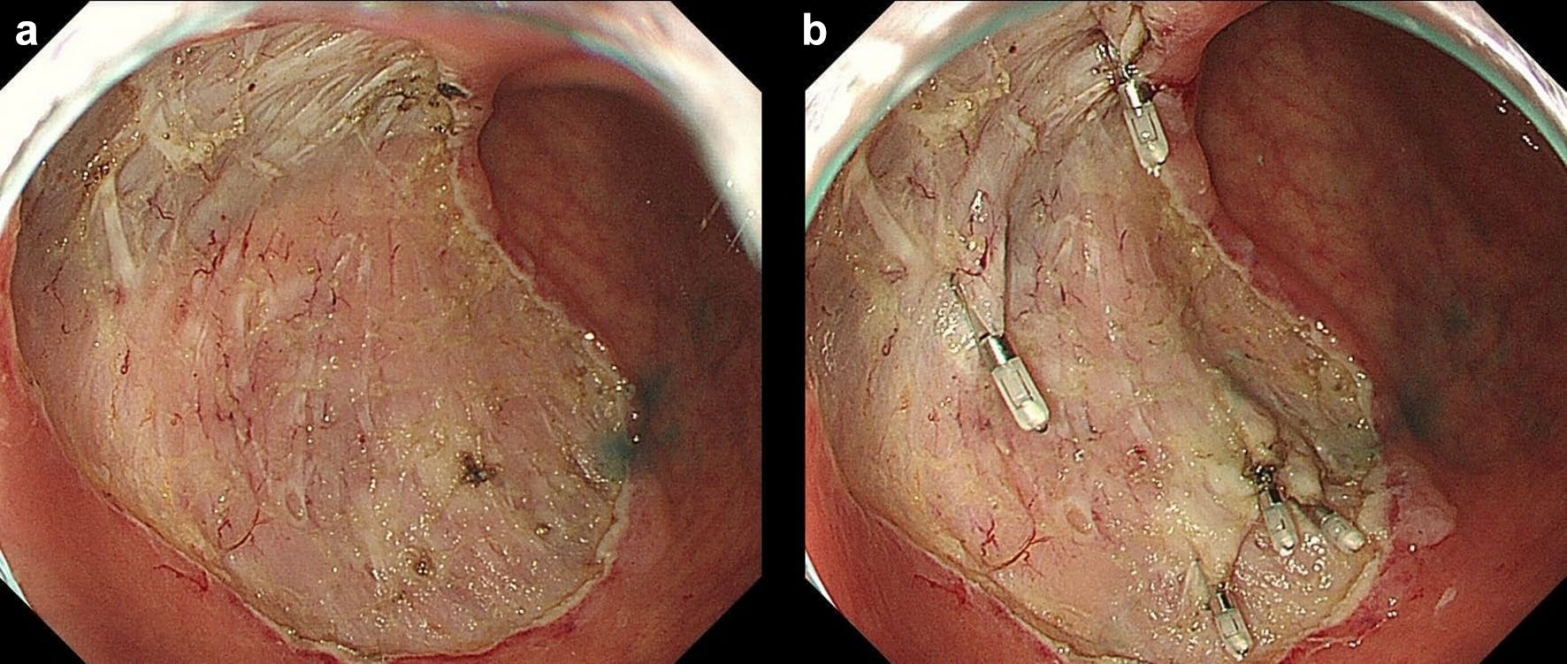
**a** Post-ESD ulcer after completion of the PEC method. *PEC* post-endoscopic submucosal dissection coagulation. **b** Post-ESD ulcer after completion of the SCC method. *SCC* search, coagulation, and clipping, *ESD* endoscopic submucosal dissection

In this study, we did not record the positional relationship of the blood vessels or perform mapping during ESD, unlike the 2C method [10]. When a good field of vision was obtained, we switched to a scope (GIF-H260Z; Olympus) on ESD completion to search for exposed blood vessels. As compared with GIF-Q260J, GIF-H260Z is more suitable for observation, as the number of pixels in GIF-H260Z is four times higher and the guide for light quantity is 1.3 times higher than those in GIF-Q260J.Blood vessels in the stomach anatomically pass through the muscle layer, and the blood vessel branches form a network in the submucosal layer [13]. Coagulation was performed at the sites where the ulcer floor was coagulated during surgery and at the perforating branches of blood vessels in the muscle layer, and then, hemostatic clips (HX-610-135; Olympus) were placed (Figs. 1a, b, 2a, b).

### Delayed bleeding

Delayed bleeding was defined as clinical post-ESD bleeding with hematemesis, melena, or anemia progression that required endoscopic examination and an additional endoscopic examination aside from the one scheduled during second-look endoscopy.

### Management of antithrombotic agents

In all patients receiving oral antithrombotic agents, the risk of thrombosis was evaluated by a cardiovascular doctor before ESD to investigate the possibility of temporarily stopping administration of the antithrombotic agents. The number of days of temporary drug discontinuation and the methods for heparin replacement were decided in accordance with the Japan Gastroenterological Endoscopy Society Guidelines. We obtained written informed consent regarding bleeding and thrombosis from all patients taking antithrombotic agents [14].

### Statistical analysis

For descriptive statistics, continuous variables are presented as mean ± standard deviation or median (range), whereas discrete variables are presented as frequency and proportion. The normality of continuous variable distributions was assessed using the Shapiro–Wilk test. Multivariate logistic regression analysis with forward stepwise regression was performed to identify the significant independent factors associated with delayed bleeding. All statistical analyses were performed using SPSS version 20.0 (IBM Corp, Armonk, NY, USA). A *P* value < 0.05 was considered significant.

### Propensity-score-matched analysis

To reduce the effect of procedure-selection bias, we performed propensity-score matching (PSM) of SCC procedure selection. The scores were calculated as the log-odds obtained by the logistic regression model, with response variables including the PEC or SCC groups. Explanatory variables consisted of location, specimen size, antithrombotic agents, procedure time, and heparin placement. Matching was performed using a 1:1 matching protocol with nearest-neighbor matching without replacement. The propensity score was rounded from three to two decimal places. Matching was performed using two decimal places for the SCC and PEC groups.

After propensity-score matching, we compared the delayed bleeding rates between the PEC and SCC groups. A two-sided *P* value < 0.05 in the Chi square test or Fisher’s exact test was considered statistically significance. PSM was performed using SPSS version 20.0 (IBM Corp, Armonk, NY, USA).

## Results

The study included 438 patients (323 male and 115 female patients). The baseline characteristics of the patients are presented in Table 1. The median (range) patient age was 73 (41–94) years, and the median (range) resected specimen length and tumor length were 35 (15–105) and 15 (0–100), respectively. In addition, the median (range) procedure time was 84 (10–461) min. Intraoperative perforation was noted in 7 of the 438 patients (1.6%), and postoperative stenosis was noted in 4 of the 438 patients (0.9%). All affected patients showed improvement with conservative or endoscopic treatment only, and no patient required surgery. No patient had delayed perforation/bleeding associated with the SCC method (Table 1).

**Table 1:** Patient, lesion, and procedure characteristics

Variable	Category	Distribution
Patient characteristics		
	Number, *n*	438
Age (years)		
	Median (range)	73 (41–94)
Sex, *n* (%)		
	Male	323 (73.7)
	Female	115 (26.3)
Antithrombotic agents, *n* (%)		
	None	360 (82.2)
	Warfarin	9 (2.1)
	DOAC	12 (2.7)
	Antiplatelet therapy	47 (10.7)
	Others	10 (2.3)
Lesion characteristics		
Location, *n* (%)		
	Upper	183 (41.8)
	Middle	191 (43.6)
	Lower	64 (14.6)
Specimen size (mm)		
	Median (range)	35 (15–105)
Specimen size, *n* (%)		
	≥ 40 mm	165 (37.7)
	> 40 mm	273 (62.3)
Histological type, *n* (%)		
	Adenocarcinoma	401 (91.5)
	Adenoma	27 (6.2)
	Others	10 (2.3)
Depth, *n* (%)		
	Intramucosa	390 (89)
	Submucosa	48 (11)
Tumor size (mm)		
		15 (0–100)
Macroscopic type, *n* (%)		
	Depressed	213 (51.4)
	Non-depressed	225 (48.6)
Ulceration, *n* (%)		
	Negative	404 (92.2)
	Positive	34 (7.8)
HM, *n* (%)		
	Negative	435 (99.3)
	Positive	3 (0.7)
VM, *n* (%)		
	Negative	432 (98.6)
	Positive	6 (1.4)
ly, *n* (%)		
	Negative	427 (97.5)
	Positive	11 (2.5)
v, *n* (%)		
	Negative	430 (98.2)
	Positive	8 (1.8)
Procedure characteristics		
Procedure time (min)		
	Median (range)	84 (10–461)
Time of SCC (s)		
	Median (range)	297 (38–1075)
Heparin placement		
	Without	424 (96.8)
	With	14 (3.2)
Second-look endoscopy		
Additional coagulation		
	Without	421 (96.1)
	With	17 (3.9)
Additional clipping		
	Without	429 (97.7)
	With	9 (2.3)
Perforation during ESD, *n* (%)		
	Negative	431 (98.4)
	Positive	7 (1.6)
Post-ESD stenosis, *n* (%)		
	Negative	434 (99.1)
	Positive	4 (0.9)

*DOAC* direct oral antithrombotic agent, *HM* horizontal margin, *VM* vertical margin, *ly* lymphatic invasion, *v* venous invasion, *ESD* endoscopic submucosal dissection, *SCC* search, coagulation, and clipping

According to the clinical pathway, the second-look endoscopy was performed for all cases. No patient presented bleeding in the stomach during second-look endoscopy. For patients in whom an exposed vessel was suspected or those who presented with oozing following flushing during the second look, of the 222 patients in the SCC group, 7 and 6 patients underwent additional cauterization and additional clipping, respectively, and of the 216 patients in the PEC group, 10 and 3 patients underwent additional cauterization and additional clipping, respectively (Tables 1, 2).

**Table 2:** Comparison of the PEC and SCC groups

Variable	PEC group	SCC group	*P* value
	(*n* = 216)	(*n* = 222)	
Delayed bleeding, *n* (%)			
Negative	200 (92.8)	217 (97.4)	0.013^a^
Positive	16 (7.2)	5 (2.6)	
Number of clips, *n*			
Mean (SD)	0	4.87 (2.28)	
Median (range)	0	4.0 (1–12)	< 0.001^b^
Age (years)			
Median (range)	73.0 (46–91)	74.0 (41–94)	0.688^b^
Sex, *n* (%)			
Male	153 (70.8)	131 (59.0)	0.172^a^
Female	63 (29.2)	91 (41.0)	
Antithrombotic agents, *n* (%)			
None	173 (80.1)	187 (84.2)	0.279^a^
Warfarin	6 (2.8)	3 (1.4)	
DOAC	6 (2.8)	6 (2.7)	
Antiplatelet therapy	23 (10.6)	24 (10.8)	
Others	8 (3.7)	2 (0.9)	
Delayed bleeding in patients who received antithrombotic agents (%)			
	11.6	5.7	0.31^c^
Location, *n* (%)			
Upper	97 (44.9)	86 (38.7)	0.409^a^
Middle	88 (40.7)	103 (46.4)	
Lower	31 (14.4)	33 (14.9)	
Specimen size (mm)			
Median (range)	32 (15–90)	35(16–105)	0.013^b^
Specimen size, *n* (%)			
≥ 40 mm	142 (65.7)	131 (59.0)	0.146^b^
> 40 mm	74 (34.3)	91 (41.0)	
Tumor size (mm)	12 (0–65)	15 (0–100)	< 0.001^b^
Ulceration, *n* (%)			
Negative	209 (99.7)	195 (87.9)	< 0.001^a^
Positive	7 (0.30)	27 (12.1)	
Procedure time (min)			
Median (range)	65.5 (9–357)	84.0 (10–461)	0.01^b^
Heparin placement			
Without	212 (96.3)	216 (97.3)	0.305^a^
With	8 (3.7)	6 (2.7)	
Second-look endoscopy			
Additional coagulation			
Without	206 (95.4)	215 (96.8)	0.672^a^
With	10 (4.6)	7 (3.2)	
Additional clipping			
Without	213 (97.7)	216 (97.3)	0.400^a^
With	3 (2.3)	6 (2.7)	
Perforation during ESD, *n* (%)			
Negative	213 (98.6)	218 (98.2)	0.676^a^
Positive	3 (1.4)	4 (1.8)	
Post-ESD stenosis, *n* (%)			
Negative	214 (99.1)	216 (99.1)	0.978^a^
Positive	2 (0.9)	2 (0.9)	

*PEC* post-ESD coagulation, *SCC* search, coagulation, and clipping, *SD* standard deviation, *DOAC* direct oral antithrombotic agent, *ESD* endoscopic submucosal dissection

^a^Chi-square test

^b^Mann–Whitney *U* test

^c^Fisher’s exact test

Of the 438 patients, 216 underwent PEC (PEC group) and 222 underwent SCC (SCC group). The comparison of characteristics between the PEC and SCC groups is presented in Table 2.

A total of 21 patients developed delayed bleeding. Delayed bleeding was significantly less common in the SCC group than in the PEC group (2.6% [5/222] vs. 7.2% [16/216]; *P* = 0.013). The specimen length was significantly larger in the SCC group than in the PEC group (35 [16–105] mm vs. 32 [15–90] mm; *P* = 0.013). In addition, the tumor length was significantly larger in the SCC group than in the PEC group (15 [0–100] mm vs. 12 [0–65] mm; *P* < 0.001). The procedure time was significantly longer in the SCC group than in the PEC group (84.0 [10–461] min vs. 65.5 [9–357] min; *P* = 0.01) (Table 2).

Antithrombotic therapy was being used by 43 patients from the PEC group and 35 patients from the SCC group, and there was no significant difference between the groups (*P* = 0.279). The delayed bleeding rates among patients treated with antithrombotic therapy were 11.6% (5/43) in the PEC group and 2.9% (1/35) in the SCC group, and there was no significant difference between the groups (*P* = 0.15), although the rate was lower in the SCC group (Table 2).

To identify the independent factors associated with delayed bleeding after gastric ESD, we conducted a multivariate analysis with a logistic regression model using all the variables in Table 1 as candidate factors. The SCC method was found to be a significant independent factor for the prevention of delayed bleeding [odds ratio (OR) = 0.289; 95% confidence interval (CI) 0.104–0.804; *P* = 0.017] (Table 3).

**Table 3:** Multivariate analysis for the factors associated with delayed bleeding after ESD

Variable	Estimated regression coefficient	SE	OR (95% CI)	*P* value^a^
SCC method	− 1.24	0.522	0.289 (0.104–0.804)	0.017

*ESD* endoscopic submucosal dissection, *SE* standard error of the regression coefficient, *OR* odds ratio, *CI* confidence interval, *SCC* search, coagulation, and clipping

^a^Wald test

We performed propensity-score matching to reduce the procedure-selection bias in terms of location, specimen size, antithrombotic agents, procedure time, and heparin placement to determine whether SCC should be performed. As a result, matching was performed in 156/216 patients in the PEC group and 156/222 patients in the SCC group. There was a significant difference in the incidence of bleeding in 14/156 patients and 4/156 patients in the PEC and SCC groups, respectively (*P* = 0.013). No statistically significant difference was observed in the patient characteristics of the two groups after adjustment for PSM (Table 4).

**Table 4:** Comparison of the PEC and SCC groups after propensity-score-matching analysis

Variables	PEC (*N* = 156)	SCC (*N* = 156)	*P*
Delayed bleeding			
Negative	142 (92.3)	152 (97.3)	0.0130^a^
Positive	14 (7.7)	4 (2.6)	
Age	72 (46–91)	75 (41–94)	0.0670^b^
Sex			
Male	112 (71.8)	118 (75.6)	0.2600^c^
Female	44 (28.2)	38 (24.4)	
Antithrombotic agents			
None	130 (83.3)	131 (84.0)	0.6900^c^
Warfarin	2 (1.3)	2 (1.3)	
DOAC	6 (3.8)	2 (1.3)	
Antiplatelet therapy	17 (10.9)	20 (12.8)	
Others	1 (0.6)	1 (0.6)	
Location			
Upper	64 (41.0)	63 (40.4)	0.7040^c^
Middle	73 (46.8)	69 (44.2)	
Lower	19 (12.2)	24 (15.4)	
Specimen size	35 (15–90)	35 (16–88)	0.7650^b^
Specimen size 40			
< 40	99 (63.5)	99 (64.6)	0.5470^c^
≥ 40	57 (36.5)	57 (35.4)	
Tumor size	12 (0–65)	15 (0–77)	0.0607^b^
Procedure time	72.0 (9–357)	80.0 (10–350)	0.2890^b^
Heparin placement			
Without	152 (96.2)	153 (98.1)	0.2510^a^
With	6 (3.8)	3 (1.9)	

*PEC* post-ESD coagulation, *SCC* search, coagulation, and clipping, *SD* standard deviation, *DOAC* direct oral antithrombotic agent, *ESD* endoscopic submucosal dissection

^a^Fisher’s exact test

^b^Mann–Whitney *U* test

^c^Chi-square test

Of the 21 patients who developed delayed bleeding, 8 had bleeding during hospitalization for ESD and 13 had bleeding after discharge and were subsequently readmitted. Seven patients required blood transfusions, and all patients required hospitalization. The mean cost at the time of readmission for delayed bleeding was 3964 USD/438,821 JPY (Table 5).

**Table 5:** Details of the 21 patients with delayed bleeding

Patient	Procedure	Timing of delayed bleeding	Transfusion	Readmission	Cost of readmission	Antithrombotic agent	Heparin placement
					(USD/JPY)		
1	PEC	Day 5				Warfarin	+
2	PEC	Day 15		+	(2542/279,780)	DOAC	+
3	PEC	Day 15	+	+	(6531/718,820)	DOAC, aspirin	
4	PEC	Day 14		+	(5366/590,610)	DOAC	
5	PEC	Day 7		+	(5254/578,270)		
6	PEC	Day 11		+	(2814/309,790)		
7	PEC	Day 5					
8	PEC	Day 7				DOAC	+
9	PEC	Day 6					
10	PEC	Day 11	+	+	(5679/625,110)		
11	PEC	Day 1					
12	PEC	Day 1					
13	PEC	Day 6	+	+	(2134/234,910)		
14	PEC	Day 10		+	(2566/282,480)		
15	PEC	Day 0 (6 h)					
16	PEC	Day 2					
17	SCC	Day 15	+	+	(4386/482,730)		
18	SCC	Day 10	+	+	(2971/327,010)	Dual-antiplatelet therapy	
19	SCC	Day 7		+	(2556/281,370)		
20	SCC	Day 10	+	+	(4749/522,700)		
21	SCC	Day 9	+	+	(4280/471,090)		

*PEC* post-endoscopic submucosal dissection coagulation, *DOAC* direct oral antithrombotic agent, *SCC* search, coagulation, and clipping

## Discussion

Delayed bleeding after ESD is a very invasive complication and should be prevented [4]. PEC, which was reported by Takizawa et al., is widely used as a simple and inexpensive method to prevent bleeding [3]. Mukai et al. reported good results with clipping after PEC (2C method) [10]. In the present study, 7.2% of patients in the PEC group and 2.6% of patients in the SCC group had delayed bleeding, and the rate was significantly lower in the SCC group. Furthermore, multivariate analysis indicated that the SCC method was an independent factor for the prevention of delayed bleeding. The results of the report by Mukai et al. and the present report suggest that the addition of clipping after PEC has an additive effect in the prevention of delayed bleeding [10]. The 2C method involves the laborious task of mapping during surgery; however, considering the small number of clips used, this method might be superior in terms of cost. In this study, there were no adverse events associated with the addition of clips in the SCC method. In addition, in the report by Mukai et al., there were no complications associated with the addition of clips in the 2C method, and it was reported that only 0.9% of clips remain at day 60 after ESD, suggesting that the possibility of long-term complications is extremely low [10]. Thus, both methods can be considered simple and safe.

Although the incidence of bleeding in the SCC group in our study (5/222 patients; 2.3%) was lower than that in previous studies (3–5.5%), the incidence of bleeding tended to be higher in the PEC group (16/216 patients; 7.4) [3–9]. There was no significant difference in bleeding risk factors, such as oral administration of anticoagulant agents (78/438 patients; 17.8%) and diameter of the resection (35; 15–105), between the results of our study for the PEC group and those of previous studies. The number of patients in previous studies was higher (485–1814 patients) than that in the PEC group of our study (216 patients). Although it was difficult to identify the cause of a higher incident of bleeding because of the smaller sample, 5 of the 12 patients (6 and 6 patients who received warfarin and DOAC orally, respectively, in the PEC group) experienced secondary hemorrhage (41.6%); 3 of these 5 patients had undergone heparin placement (Table 5). Thus, failure to prevent bleeding in patients receiving anticoagulant agents orally is considered to be one of the causes for a higher incidence of bleeding.

It is essential to perform dissection immediately above the muscle layer to identify blood vessels penetrating the muscle layer, which have a high risk of delayed bleeding. However, it is difficult to determine if sufficient cauterization to the deep layer of the muscle has been achieved with cauterization using hemostatic forceps. Blood vessels that are not fully cauterized in the deep part of the muscle may start to bleed. Considering that the ulcer floor immediately after ESD is soft, placement of additional clips is thought to help prevent delayed bleeding after ESD through hemostasis of blood vessels that are not fully cauterized in the deep part of the muscle. The best time for clipping is thought to be immediately after ESD, as white material adheres to the site a certain amount of time after ESD, which makes it difficult to observe exposed blood vessels, and the ulcer floor hardens over time, which makes it difficult to add clips.

With regard to the cost-effectiveness of the SCC method, 1 clip costs 7.5 USD/825 JPY, and in this study, 4.9 clips were required on average. Thus, the cost per patient was 36.7 USD/4,042 JPY. If we consider 100 patients, the price of the clips becomes 3,670 USD/404,200 JPY. In this study, among the 21 patients with delayed bleeding, 13 required re-hospitalization and 7 required blood transfusions. The average hospitalization cost among the 13 patients who required re-hospitalization was 3,964 USD/438,821 JPY. If we presume a bleeding rate of 2.3% in the SCC group and 5% in the PEC group, among 100 patients, there would be 2.3 and 5 patients with delayed bleeding in the respective groups [3–9]. If the number of patients with delayed bleeding requiring re-hospitalization is reduced by even one, the re-hospitalization cost of 3,964 USD/438,821 JPY will be saved. Thus, the SCC method is cost-effective, particularly if we consider the invasiveness of the procedure.

Patients receiving antithrombotic therapy have been reported to be at high risk of delayed bleeding after gastric ESD, with a bleeding rate of 21–38% [15–18]. Recent reports have indicated the effectiveness of polyglycolic acid (PGA) sheets (Neoveil; Gunze, Kyoto, Japan) and fibrin glue (Beriplast P Combi-Set; CSL Behring Pharma, Tokyo, Japan) for preventing delayed bleeding after ESD in patients receiving antithrombotic therapy [19–21]. Kawata et al. reported a method of covering post-ESD ulcers with PGA in patients who underwent ESD while receiving antithrombotic agents. Without its discontinuation, they mentioned that the bleeding rate was significantly lower in the PGA group (5.8% [3/52]) than in the control group (20.8% [11/53]) [21]. In the present study, the rate of delayed bleeding among patients receiving antithrombotic therapy was 11.6% (5/43) in the PEC group and 2.9% (1/35) in the SCC group (*P* = 0.15), indicating a favorable outcome with a lower rate of bleeding in the SCC group. Only 1 patient on dual-antiplatelet therapy had delayed bleeding in the SCC group. Kawata et al. performed a study in which patients without discontinuation of antithrombotic agents were at higher risk of bleeding, and reported a significant difference between them and the control group. It was suggesting that the SCC method may help prevent delayed bleeding in patients taking antithrombotic agents, similar to PGA sheets.

Previous studies reported that the location of the main seat of the tumor in the L region, length of the tumor, length of the resected specimen, and duration of the procedure were independent risk factors for delayed bleeding after ESD [5–7, 9, 22]. In this study, although the length of the tumor, length of the resected specimen, and duration of the procedure were significantly greater in the SCC group than in the PEC group, there were significantly fewer cases of delayed bleeding in the SCC group than in the PEC group. In addition, in multivariate analysis, the SCC method was found to be an independent factor for the prevention of delayed bleeding. Moreover, on comparing the SCC and PEC groups following propensity-score matching for correction of the selection bias to determine whether SCC is to be performed, the incidence of secondary hemorrhage was found to be significantly lower in the SCC group. Although this was a retrospective analysis, the results from univariate analysis, multivariate analysis, and PSM suggest that SCC might prevent secondary hemorrhage in patients who undergo gastric ESD. To our knowledge, the present study is the first to indicate that clipping of blood vessels at the ulcer floor is very effective for preventing delayed bleeding after gastric ESD in both univariate and multivariate analyses.

The limitations of this study were its retrospective design and patient recruitment from a single center. It is essential to confirm the efficacy of the SCC method in a prospective, randomized controlled trial.

## Conclusion

We found that the SCC method was more effective than conventionally used PEC for preventing delayed bleeding. The SCC method is a simple, inexpensive, safe, and effective approach for preventing delayed bleeding after gastric ESD.
