# Correction to: The search, coagulation, and clipping (SCC) method prevents delayed bleeding after gastric endoscopic submucosal dissection

**DOI:** 10.1007/s10120-018-0886-y

**Published:** 2018-10-23

**Authors:** Motoi Azumi, Manabu Takeuchi, Youhei Koseki, Masaru Kumagai, Yoko Kobayashi, Masafumi Takatsuna, Aiko Yoshioka, Seiichi Yoshikawa, Tsutomu Miura, Shuji Terai

**Affiliations:** 1 Division of Gastroenterology and Hepatology, Nagaoka Red Cross Hospital, 2-297-1, Chiaki, Nagaoka, Niigata 940-2085 Japan; 2 Division of Gastroenterology and Hepatology, Graduate School of Medical and Dental Sciences, Niigata University, Niigata, Japan

## Correction to: Gastric Cancer 10.1007/s10120-018-0878-y

The article “The search, coagulation, and clipping (SCC) method prevents delayed bleeding after gastric endoscopic submucosal dissection”, written by Motoi Azumi, Manabu Takeuchi, Youhei Koseki, Masaru Kumagai, Yoko Kobayashi, Masafumi Takatsuna, Aiko Yoshioka, Seiichi Yoshikawa, Tsutomu Miura, and Shuji Terai, was originally published electronically on the publisher’s internet portal (currently SpringerLink) on 28 September 2018 without open access.

With the author(s)’ decision to opt for Open Choice the copyright of the article changed on 15 October 2018 to © The Author(s) 2018 and the article is forthwith distributed under the terms of the Creative Commons Attribution 4.0 International License (http://creativecommons.org/licenses/by/4.0/), which permits use, duplication, adaptation, distribution and reproduction in any medium or format, as long as you give appropriate credit to the original author(s) and the source, provide a link to the Creative Commons license and indicate if changes were made.

In addition, the given name and surname were swapped in the author group. The corrected list of author names is presented above.

The original article has been corrected.

